# Simple and Cost-Effective Restriction Endonuclease Analysis of Human Adenoviruses

**DOI:** 10.1155/2014/363790

**Published:** 2014-03-09

**Authors:** Arun Kumar Adhikary, Nozomu Hanaoka, Tsuguto Fujimoto

**Affiliations:** ^1^Unit of Microbiology, Faculty of Medicine, AIMST University, Semeling, 08100 Bedong, Kedah Darul Aman, Malaysia; ^2^Infectious Disease Surveillance Center, National Institute of Infectious Diseases, 1-23-1 Toyama, Shinjuku-ku, Tokyo 162-8640, Japan

## Abstract

Restriction endonuclease analyses (REAs) constitute the only inexpensive molecular approach capable of typing and characterizing human adenovirus (HAdV) strains based on the entire genome. However, the application of this method is limited by the need for time-consuming and labor-intensive procedures. We herein developed a simple and cost-effective REA for assessing HAdV. The method consists of (1) simple and cost-effective DNA extraction, (2) fast restriction endonuclease (RE) digestion, and (3) speedy mini agarose gel electrophoresis. In this study, DNA was isolated according to the kit-based method and 21.0 to 28.0 **μ**g of viral DNA was extracted from prototypes (HAdV-1, HAdV-3, HAdV-4, and HAdV-37) in each flask. The amount of DNA ranged from 11.4 to 57.0 **μ**g among the HAdV-3 (*n* = 73) isolates. The obtained viral DNA was found to be applicable to more than 10 types of REAs. Fast-cut restriction endonucleases (REs) were able to digest the DNA within 15 minutes, and restriction fragments were easily separated via horizontal mini agarose gel electrophoresis. The whole procedure for 10 samples can be completed within approximately six hours (the conventional method requires at least two days). These results show that our REA is potentially applicable in many laboratories in which HAdVs are isolated.

## 1. Introduction

Human adenoviruses (HAdVs) are divided into seven species, A (HAdV-A) through G (HAdV-G), based on various biological and morphological criteria, nucleic acid characteristics, and homologies [1]. Approximately one-third of HAdVs are associated with human diseases, being estimated to cause 8% of clinically relevant viral diseases globally. Common adenoviral diseases include respiratory infections in children and military recruits, infantile gastroenteritis, and ocular infections among healthy individuals. Less frequently, these pathogens can cause urinary tract infections, myocarditis, meningoencephalitis, and acute hemorrhagic cystitis [2]. Meanwhile, in neonates and immunocompromised individuals, HAdVs have been reported to cause fulminant fatal pneumonia, hepatitis, and/or encephalitis [3, 4]. Genetically variable strains are present within a type designated as the genome type or DNA variant [5]. The genome type is determined based on a panel of a restriction endonuclease analysis (REA) of the viral genome. The site of cleavage of DNA by a restriction endonuclease is sequence dependent, and the presence of mutations at potential cleavage sites, insertions, and deletions anywhere in the genome results in different patterns of fragments when separated on agarose gel, a phenomenon termed restriction fragment length polymorphism [6]. The profile of DNA fragments visualized via gel electrophoresis can be compared to other published profiles of adenovirus isolates in order to designate the genome type [7]. REA is currently the only inexpensive molecular approach capable of typing and characterizing HAdV strains based on the entire genome.

Two systems for naming HAdV genome type/DNA variants are currently in use. In one nomenclature system, the prototype strain is abbreviated as “p,” while the other strains were designated as a, b, c, and so forth in order of discovery [5, 8]. In other classification systems, numerical codes for multiple restriction enzymes are used to denominate the genome type. In this method restriction endonucleases are displayed in alphabetical order. The prototype restriction profile is designated as 1, and each profile distinct for a given endonuclease is designated as 2, 3, 4, and so forth in chronological order of each new profile [9].

Molecular epidemiological studies have been conducted using genome typing, and more than 20 genome types of HAdV-7 have been reported to date. Among them, HAdV-7 h and HAdV-7d are reported to be virulent. HAdV-7 h became a predominant genotype in South America in 1986 and has circulated in North America since 1998 [10, 11]. HAdV-7d is associated with an 18% fatality rate in Korea among infants and children who presented with clinical evidence of lower respiratory tract infections; while HAdV-3 includes 51 genome types, many of which are associated with fatal infections. For example, HAdV-3a17 exhibits a 3.6% fatality rate among pediatric patients [12]. Ten genome types of HAdV-8 have been reported. HAdV-8e circulates worldwide and is related to many outbreaks of epidemic keratoconjunctivitis, whereas HAdV-8j is a localized strain [13, 14]. Therefore, it is clear that the HAdV genome types differ in virulence, and characterizing these strains is thus both clinically and epidemiologically important.

However, genome typing methods are time consuming and labor intensive, with their success being primarily dependent upon the extraction of a fairly large amount of viral DNA. DNA can be extracted from culture fluid, virus-infected cell lines, or both. The standard protocol for DNA extraction using ultracentrifugation involves the purification of viral particles obtained from infected cells, which requires time-consuming steps, such as cell disruption, cesium chloride centrifugation, and dialysis [15]. Another widely used method, developed by Hirt, or modified Hirt's methods, consists of extracting the HAdVs from infected cell DNA without prior purification of the virions [16, 17]. In these methods, overnight NaCl precipitation of cellular genomic DNA is required. Moreover, the above methods for preparing HAdV DNA require many steps and cannot be performed within one day. Methods employing slightly quicker extraction of DNA using a 75 cm^2^ culture flask via short ultracentrifugation or concentration of the virus using a membrane filter followed by subsequent treatment with proteinase K to hydrolyze the viral proteins with final ethanol precipitation of viral DNA have been reported [18, 19]. However, the amount of DNA obtained using these methods is not sufficient for multiple REA digestion.

Considering the clinical and epidemiological importance of genome typing, we developed a one-day REA method that significantly reduces the time required to extract DNA from adenovirus-infected A549 cells using a commercial DNA extraction kit, rapid DNA digestion, and quick electrophoresis.

## 2. Materials and Methods

### 2.1. HAdV Strains

Prototype strains of HAdV-1, HAdV-3, HAdV-4, and HAdV-37 were obtained from the American Type Culture Collection (ATCC, Rockville, MD). A total of 73 HAdV-3 strains isolated over 22 years in Japan were used.

### 2.2. Viral Culture and Extraction of Viral DNA

The stock viruses were grown in A549 cells. A sample of each stock virus was further grown in the same cell line in a 25 cm^2^ tissue culture flask (Becton Dickinson). The cells were infected according to standard procedures at an approximate multiplicity of infection of 10. The cells were observed every day for cytopathic effects (CPEs). When a CPE of 80% or more was detected, the cells were dislodged via tapping and/or with a rubber policeman, and the cells with medium were collected in a 15 mL conical centrifuge tube. The tube was then centrifuged at 1,500 ×g for five minutes to form cell pellets. The supernatant was discarded, and the cells were mixed via vortexing with the remaining medium (approx. 200 *μ*L). The viral DNA was extracted using the High Pure Viral Nucleic Acid Kit (Roche). First, 200 *μ*L of binding buffer supplemented with 50 *μ*L of proteinase K (200 *μ*g/mL) was mixed well, after which the content was immediately transferred to a 1.5 mL microcentrifuge tube and incubated at 72°C for 10 minutes. Then, 100 *μ*L of binding buffer was added to the tube and mixed well. Poly A included in the kit was not used. The content was subsequently transferred to a spin column combined with the collection tube, and the column was centrifuged at 8,000 ×g for one minute then combined with a new collection tube. The old collection tube and contents were discarded. Four hundred and fifty microliters of wash buffer was added to the column and centrifuged at 8,000 ×g for one minute. The column was then combined in a new collection tube, and the collection tube and contents were discarded. Then, 450 *μ*L of washing buffer was again added to the column and centrifuged a second time at 8,000 ×g for one minute followed by 13,000 ×g for 10 seconds in order to drain out the washing buffer. The column was combined with a 1.5 mL microcentrifuge tube, and 100 *μ*L of elution buffer was added to the column and centrifuged at 8,000 ×g for one minute, after which the column was disposed. The contents remaining in the microcentrifuge tube comprised viral genomic DNA.

### 2.3. Quantitation of DNA

The concentrations of genomic DNAs were measured using a BioSpec-nano spectrophotometer (Shimadzu Corporation, Kyoto, Japan). In short, 2 *μ*L (path length: 0.7 mm) of viral DNA was mounted in sample stage, and the instrument automatically measured the DNA concentration. The purity of DNA was considered significant for an optical density ratio (OD ratio) OD 260/OD 280 of approximately 1.8 and less than 2.0. The copy numbers of randomly selected viral DNA (*n* = 5) were measured using real-time PCR [20] after dilution (×100). To confirm the quality of the extracted DNA, 1.0 *μ*L of the extracted DNA was electrophoresed on 1% horizontal submerged agarose mini gel. The electrophoresis was performed in TAE buffer at 100 V for 50 minutes (Mupid, Advance, Tokyo, Japan).

### 2.4. DNA Restriction Endonuclease Analysis and Agarose Gel Electrophoresis

Both usual RE and RE fast digestion of HAdV-1p, HAdV-3p, HAdV-4p, and HAdV-37p were performed with usual REs and high fidelity (HF) REs, such as* BamHI*,* BglII*, and* HindIII* (New England Biolabs), which have the power to complete digestion within 5–15 minutes. A total of 1 *μ*g of DNA was incubated with 10 units of fast digest RE in 20 *μ*L of reaction mixture at an appropriate temperature and time (recommended for each RE). The fragmented DNA and molecular weight markers are loaded in separate wells on 1.2% horizontal submerged agarose mini gel (size, 106 mm (*W*) × 60 mm (*L*)) made with a thicker comb (6 mm × 12 well), and electrophoresis was performed in TAE buffer at 50 V for one hour and 50 minutes (Mupid). The electrophoresis machine was cooled on ice or in a cooler (Cosmo-bio, Tokyo, Japan) to keep the buffer cool during electrophoresis. After electrophoresis, the DNA in the gel was stained for 30 minutes in GelRed (Biotium, Hayward, CA) solution made in TAE buffer. Lambda DNA-HindIII digest marker (New England Biolabs) photographs were taken under UV light. For electrophoresis, a prototype strain was always added as a control.

## 3. Ethical Considerations

This study did not use clinical samples but rather isolates obtained using anonymous information that cannot be associated with the individual patient. Therefore, the study protocol did not require ethics committee approval. For HAdV isolation, Ethical Committee has approved the experiments (ID25-35).

## 4. Results 

### 4.1. Viral Culture and Extraction of Viral DNA

**Figure 1:**
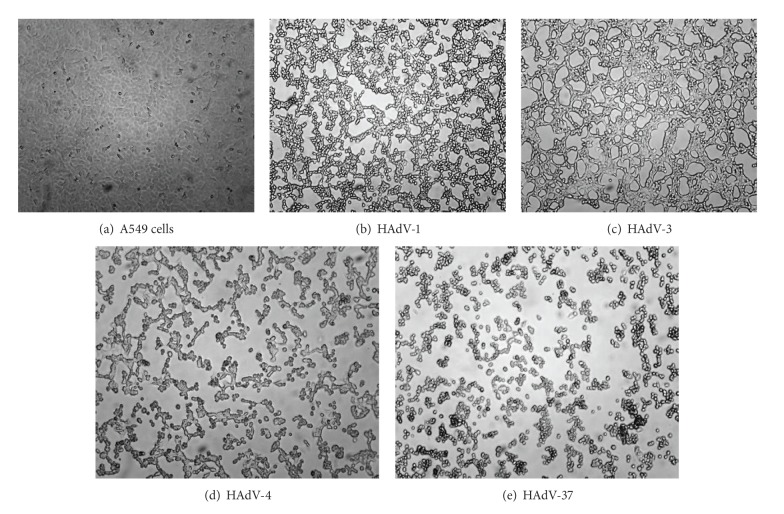
Typical HAdV CPE. (a) Noninfected A549 cells, (b) A549 cells infected with HAdV-1, (c) A549 cells infected with HAdV-3, (d) A549 cells infected with HAdV-4, and (e) A549 cells infected with HAdV-37.

Mixing the cell pellets via vortexing is important before adding the binding buffer (6 M guanidine-HCl, 10 mM Tris-HCl, 10 mM urea, 20% Triton X-100 (*w/v*), pH4.4 (+25°C)). If the binding buffer is added without creating a cell suspension, a clump of cell lysate is formed, which can clog the mesh of the spin column. The entire procedure, from collection of the infected cells from a 25 cm^2^ flask to extraction of DNA, takes one hour and 30 minutes for 10 samples. The CPE of prototypes HAdV-1, HAdV-3, HAdV-4, and HAdV-37 was visible within 24 hours and included 80% of the cells within 48 hours (Figure 1). This method has been well studied for identifying the most clinically important HAdVs that grow well in cell culture and give rise to clearly visible CPEs; however, it has not been thoroughly evaluated for identifying fastidious HAdVs, such as HAdV-41.

### 4.2. Quantitation of Genomic DNA

The mean concentration of DNA among the 73 HAdV-3 isolates was 338 ± 111 ng/*μ*L (mean ± standard deviation, range 114–576 ng/*μ*L) with an OD ratio (OD 260/280) of 1.92 ± 0.04 (mean ± standard deviation, range 1.74–1.98), as expressed by the manufacturer. A clear background in the gel also suggests the extraction of a good amount of quality DNA. The extracted DNA included 2.1 × 10^10^–9.1 × 10^10^  copies/*μ*L (mean, 5.3 × 10^10^  copies/*μ*L) of viral DNA.

### 4.3. DNA Restriction Endonuclease Analysis and Agarose Gel Electrophoresis

**Figure 2:**
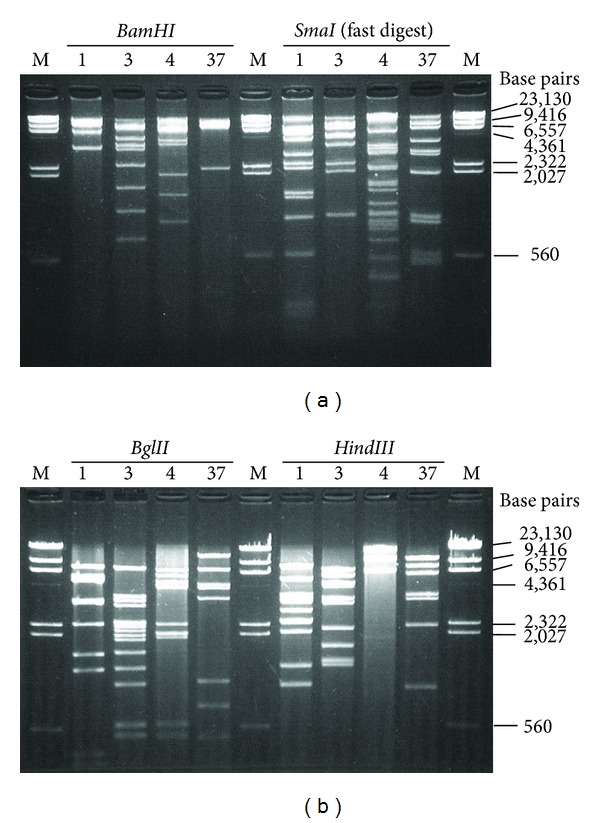
Picture of the REA. (a) REA pattern for* BamHI* and* SmaI* (fast digest). (b) REA pattern for* BglII* and* HindIII*. M shows Lambda DNA-HindIII digest marker. Numbers 1, 3, 4, and 37 show HAdV-1, -3, -4, and -37, respectively.

The* BamHI*,* BglII*,* HindIII*, and* SmaI* digestion patterns of HAdV-1p, HAdV-3p, HAdV-4p, and HAdV-37p were in agreement with previously published restriction patterns [21, 22]. The restriction pattern clearly distinguished these strains (Figures 2(a) and 2(b)). We used Mupid mini gel and big gel (125 mm (*W*) × 123 mm (*L*)) to separate the cleavage products.We found that, like big gel, mini gel can be used to separate the DNA fragments well and is adequate for use in the classification of genome types.

### 4.4. Overall Time for the Entire Procedure

Theoverall working time is dependent on the time required for DNA extraction, concentration measurement, digestion, and electrophoresis. The working time of the entire procedure is approximately six hours (Table 1).

## 5. Discussion

REA of viral DNA is established for studies of the molecular epidemiology of HAdVs. It is important to (1) understand the incidence and prevalence of different genome types, (2) identify the most prevalent genome types circulating worldwide or in a given country, (3) compare the most prevalent currently circulating HAdV genome types in order to identify possible common targets for intervention, and (4) analyze the frequency of genome types in order to identify their geographical distribution and patterns of circulation. However, performing fast REA of HAdVs poses a challenge with respect to standardizing the techniques used in various steps of the procedures, such as viral culture, lengthy DNA extraction, prolonged electrophoresis to separate DNA fragments, and the need for proper documentation of results. Occasionally, REAs must be repeated [23].

Various continuous epithelial cell lines, including HEp-2, HeLa, KB, and A549, are useful for isolating HAdVs. However, comparative studies of various cell lines have shown that the A549 cell line is very permissive, allowing for the propagation of HAdVs [24, 25]. The cells usually swell, with nuclear enlargement, followed by detachment from the surface [2]. The cytologic changes vary among the types of HAdVs, as shown in Figure 1.

The success of REA is principally dependent on the ability to extract a large amount of good quality viral DNA. In our kit-based extraction method, we have succeeded in extracting 11.4 *μ*g to 57.0 *μ*g of DNA (34 ± 11 *μ*g, mean ± SD) from 25 cm^2^ flasks, which is sufficient for analyses of multiple REAs which use 1 *μ*g per reaction. It is easier to use 25 cm^2^ flasks than 75 cm^2^ flasks to prepare confluent cell cultures and also it saves space in the CO_2_ incubator, with subsequent cost savings. In comparison to time-consuming and labor-intensive ultracentrifugation methods or Hirt's method, our procedure is appropriate for use in laboratories in which routine virology work is performed. This method also results in a significantly higher amount of DNA than other previously described DNA extraction methods [18, 19], which have limited use, as they are not cost effective and do not provide a sufficient amount of DNA. We attempted to extract DNA from 25 cm^2^ flasks using modified Hirt's method and obtained an approximately 10 times lower amount of viral genome (data not shown). This finding may be due to the many steps required in modified Hirt's method, resulting in the loss of viral DNA in each step.

Regarding the extraction of viral DNA, a visible CPE of 80% is a good result, yielding clear DNA in gel electrophoresis. However, it has not been thoroughly evaluated for fastidious HAdV, such as HAdV-41. In such case, PCR-RFLP [26] might be helpful. RE is cost effective and should not be underrepresented.

Traditionally, two hours of incubation are required for DNA cleavage using conventional endonucleases. Currently, fast-cut RE is available from various biomedical suppliers, with a similar or lower cost compared to the conventional one. These enzymes save time, as they cleave DNA within 5–15 minutes.

The ability to separate the digested DNA fragments via electrophoresis and documentation of gel images is very important for classifying the genome type. Sometimes it is difficult to distinguish important bands on these images and can be challenging to interpret the resulting restriction pattern based on comparisons with published restriction profiles [21, 27]. Therefore, we recommend the use of a prototype or known genome type of the respective isolates as a standard for quick interpretation of the results. We employed a mini horizontal agarose gel apparatus at lower voltage (50 V in this case), as it can separate the fragments clearly within two hours, which is both quick and cost effective.

Despite the clinical-epidemiological impact of REA, the number of REA-based genome typing studies is lower than expected, which may be due to the specialized and tedious nature of the procedure [7]. Therefore, PCR-based identification methods appear to be overtaking traditional serotyping and REA methods. It is important to note that PCR- and sequencing-based typing methods (molecular typing) focus on limited areas of the adenovirus genome and have the potential to overlook and mistype new HAdV variants that differ genetically in other gene regions. Recently, a genotyping method employing the complete sequence of HAdV was introduced [28]. However, this typing method is too expensive for routine study, at least in developing countries. Hence, until whole-genome sequencing (genotyping) becomes less expensive, REA will continue to have an important role in the epidemiological study of adenoviruses [29].

We have also successfully applied our REA method on different clinical isolates (data not shown) as well as on a new recombinant type. A novel HAdV-48 recombinant was isolated from eye swab of conjunctivitis patient [30] and our method was applicable to find the novel strain. We also confirmed that our REA is applicable to identify recombinant types such as HAdV-53 (AB605246) which is identical to type 22 in hexon loop region and type 8 in fiber region.

The extracted DNA exhibits adequate purity for REA and is found to be useful for direct nucleotide sequencing (data not shown). The time required to complete the extraction procedure is approximately one hour and 30 minutes for 10 samples. RE digestion and electrophoresis require an additional five hours. Fast digest RE (e.g., Takara Shuzo, Shiga, Japan) can even shorten the reaction time by more than an hour compared with the conventional method.

Our rapid REA method can significantly reduce all steps of the REA procedure, from extraction of DNA to electrophoresis of the cleavage products, thus allowing laboratories with virus culture facilities to easily perform REA of HAdVs at a lower cost. This method is potentially applicable in various research institutes as well as public health laboratories where HAdVs are routinely isolated.

## 6. Conclusion

To our knowledge, this is the first practical approach for developing a quick step-by-step REA procedure that includes extraction of good quality and adequate quantity of DNA from a small volume of infected A549 cells, fast digestion of DNA, and rapid electrophoresis of the cleavage products. The application of our newly developed REA method will allow public health and research laboratories to determine the genome type quickly and inexpensively, which will further aid epidemiological, clinical, and virological studies of HAdVs.

## Figures and Tables

**Table 1:** Overall time, including preparation time, required for the restriction endonuclease analysis (REA) of HAdVs using 10 samples.

REA steps	Time required
(1) DNA extraction from infected cells	1 hour 30 minutes
(2) Quantification of DNA of genomic DNA and electrophoresis	1 hour 15 minutes
(3) Restriction endonuclease digestion	5–15 min by fast-cut enzyme (1 hour 30 minutes by conventional enzyme)
(4) Agarose gel electrophoresis	1 hour 50 minutes
(5) Gel stain	30 minutes

Total time	Approx. 6 hours
